# Supplementary material for: Extracellular Heparan 6-O-Endosulfatases SULF1 and SULF2 in Head and Neck Squamous Cell Carcinoma and Other Malignancies
Source: Cancers (Basel). 2022 Nov 11;14(22):5553. doi: 10.3390/cancers14225553 (PMC9688903; doi:10.3390/cancers14225553)
Supplement: Supplementary file 1 [file cancers-14-05553-s001.zip › Supplementary Figures_S1-S3.pdf]

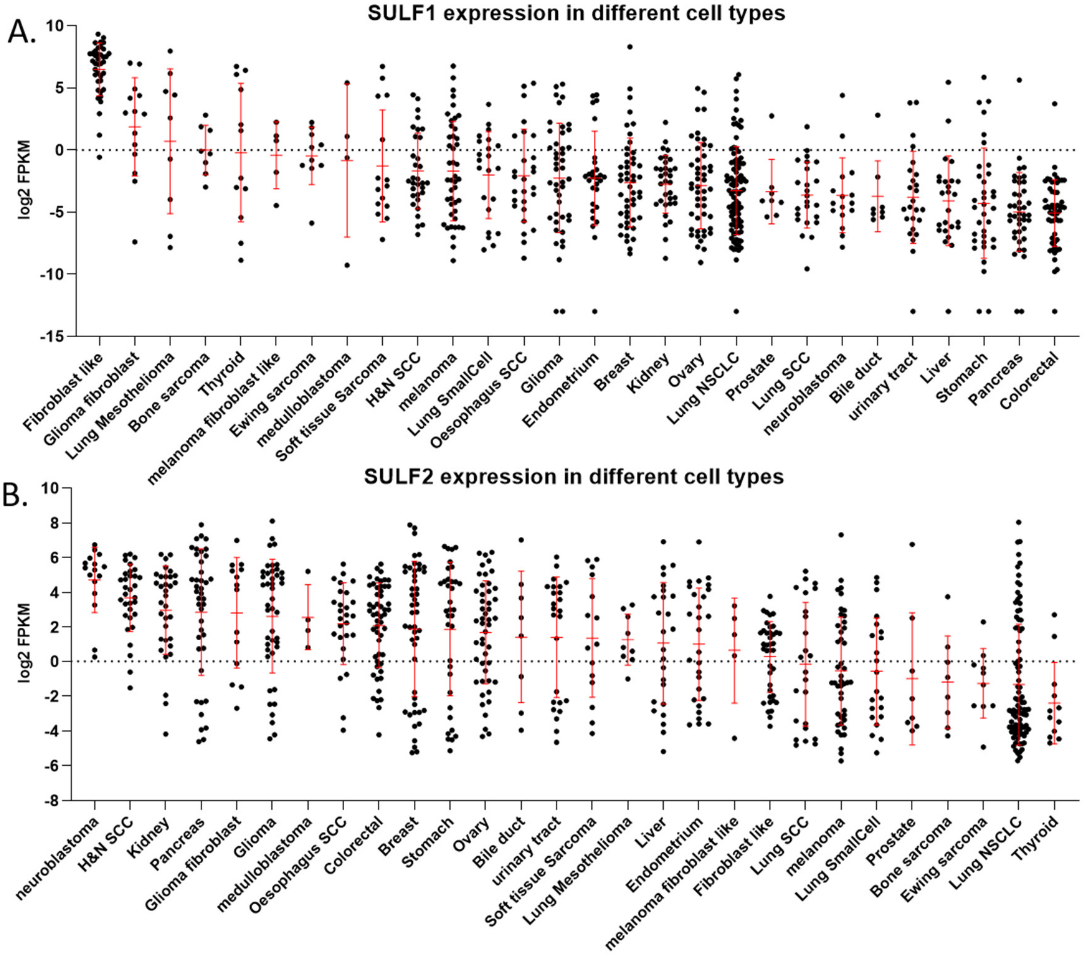

Supplemental Figure S1. A. SULF1 and B. SULF2 mRNA expression in cancer cell lines from the cancer cell-line encyclopedia (CCLE).

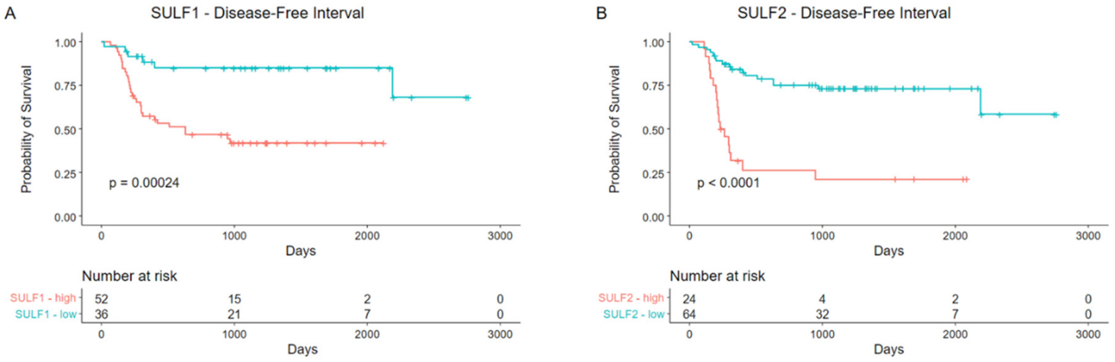

Supplemental Figure S2. Disease free survival of HNSC patients (n=88) based on the following: A. SULF1 expression; B. SULF2 expression.

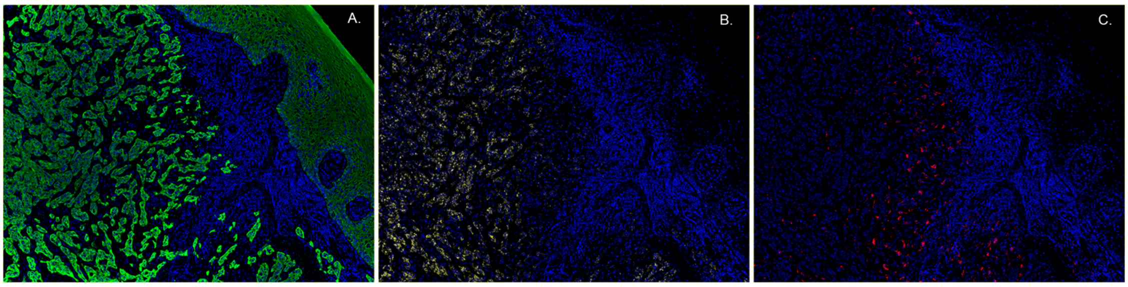

Supplemental Figure S3. mIHC and RNAScope of the OSCC tumor in Figure 6 displayed in three individual channels as follows: A. Cytokeratin (green) and DAPI (blue); B. SULF2 (yellow) and DAPI (blue); and C. SULF1 (red) and DAPI (blue).
